# *Salicornia ramosissima*: A New Green Cosmetic Ingredient with Promising Skin Effects

**DOI:** 10.3390/antiox11122449

**Published:** 2022-12-12

**Authors:** Ana Correia, Ana Margarida Silva, Manuela M. Moreira, Miguel Salazar, Jaroslava Švarc-Gajić, Tanja Brezo-Borjan, Maria de la Luz Cádiz-Gurrea, Antonio Segura Carretero, Francesca Loschi, Stefano Dall’Acqua, Cristina Delerue-Matos, Francisca Rodrigues

**Affiliations:** 1 REQUIMTE/LAQV—Instituto Superior de Engenharia do Porto, Rua Dr. António Bernardino de Almeida, 431, 4249-015 Porto, Portugal; 2 Agro-On/RiaFresh—Verduras da Ria Formosa, Sítio do Besouro, 8005-421 Faro, Portugal; 3 Faculty of Technology, University of Novi Sad, Bulevar cara Lazara 1, 21000 Novi Sad, Serbia; 4 Department of Analytical Chemistry, Faculty of Sciences, University of Granada, Fuentenueva s/n, E-18071 Granada, Spain; 5 Department of Pharmaceutical and Pharmacological Sciences, University of Padova, Via Marzolo 5, 35121 Padova, Italy

**Keywords:** *Salicornia ramosissima*, subcritical water extraction, cosmetic ingredient, eco-friendly extraction, skin permeation

## Abstract

This study aims to validate a new cosmetic ingredient from *Salicornia ramosissima* S J. Woods through in vitro and ex vivo assays. The halophyte extracts were obtained by subcritical water extraction (SWE) at different temperatures (110, 120, 140, 160 and 180 °C). The antioxidant/radical scavenging activities and the phenolic profile were screened for all extracts. The optimal extract was assessed in keratinocytes and fibroblasts, while permeation assays were performed in Franz cells. The inhibitory activity of hyaluronidase and elastase was also evaluated. The sample extracted at 180 °C presented the highest phenolic content (1739.28 mg/100 g of dry weight (dw)). Despite not being efficient in the sequestration of ABTS^•+^, this extract scavenged the DPPH^•^ (IC_50_ = 824.57 µg/mL). The scavenging capacity of superoxide (O_2_^•−^) and hypochlorous acid (HOCl) was also considerable (respectively, IC_50_ = 158.87 µg/mL and IC_50_ = 5.80 µg/mL). The cell viability assays confirmed the absence of negative effects on keratinocytes, while the fibroblasts’ viability slightly decreased. The ex vivo permeation of rutin, quercetin and syringic acid after 24 h was, respectively, 11, 20 and 11%. Additionally, the extract showed a good elastase and hyaluronidase inhibitory activity. The results obtained support the *S. ramosissima* bioactivity as a cosmetic ingredient.

## 1. Introduction

Cosmetics represent a class of products used in personal and skin care. In recent decades, the cosmetic industry has been continuously growing, being one of the biggest and successful industries worldwide. According to analysts, it is expected that cosmetic ingredients extracted from natural matrixes will be worth USD 120 million by 2022 [1], leading to the search for new ingredients. Despite that, in recent years consumers are more aware of environmental and sustainability issues, paying attention to a product’s origins and production processes as well as ecological implications and safeness. Therefore, the preference for natural bioactive compounds, which are biodegradable and have non-toxic effects, has been growing relative to synthetic ones. Based on that, the search for natural ingredients is one of the principal targets of the cosmetic industry [2]. Therefore, it is important to search for new natural raw materials rich in bioactive compounds and, simultaneously, extract them through eco-friendly procedures, leading to low environmental impacts, while meeting the consumers’ expectations. Since consumers correlate botanicals with safety, due to their natural origin, the preference for bioactive plant compounds has been aroused [3].

Salicornia is distributed worldwide in temperate and subtropical habitats, excluding South America and Australia [4]. According to James J. Woods, the species *Salicornia ramosissima*, popularly known as green samphire, is included in the species aggregate *S. europea*, being a halophyte widely distributed in the salt marshes of the Iberian Peninsula. *S. ramosissima* is a good source of minerals, such as sodium, magnesium, potassium, and calcium [5], as well as proteins, amino-acids, and vitamins [6], being consumed in northern European countries, fresh or cooked [5]. Notwithstanding being consumed, halophytes remain poorly characterized in terms of their bioactive compounds and biological properties, having antioxidants [7] and lipid compounds with a high nutritional value owing to the presence of essential fatty acids [8], and phospholipids and glycolipids with demonstrated bioactive properties [9,10]. However, to obtain the plant’s bioactive compounds, the selection of the best extraction techniques is still a key step. Current conventional extraction techniques, such as Soxhlet extraction and maceration, usually require higher organic hazardous solvent usage and long extraction times, presenting low selectivity and extraction yields [11]. These disadvantages fortified the diversification and development of new innovative green extraction techniques, also called clean techniques, such as subcritical water extraction (SWE), ultrasound-assisted extraction (UAE), microwave-assisted extraction (MAE), extrusion, and supercritical fluid extraction (SFE), that aim to overcome the drawbacks of the conventional extraction techniques [11,12] by reducing the use of energy and the extraction time, while increasing extractive yields and being significantly less harmful to the environment [11,12,13]. SWE, also known as pressurized hot water extraction, is an environmentally friendly technique that has been increasingly used as an alternative to traditional extraction methods [14,15]. SWE is characterized by the employment of subcritical water, which refers to liquid water at temperatures and pressures below its critical point (T_C_ = 374.15 °C, Pc = 22.1 Mpa), but above 100 °C (water boiling point), under sufficient pressure to maintain its liquid state (normally 10 to 100 bar, or about 1 to 10 MPa) [16]. The subcritical water pressure must be higher than the vapor pressure, to keep water in its liquid state. SWE applications range from the isolation of numerous polar to low-polarity compounds, namely phenolic compounds, essential oils, polysaccharides and protein [17]. Apart from being a green method, the SWE time is significantly shorter when compared to classical extraction techniques, maintaining the high quality of extraction, with a lower number of operation steps. Furthermore, lower surface tension and viscosity may improve efficiency, whereas broad possible temperature range allows the selective extraction of different chemical classes [17]. Nevertheless, this technique also presents a few disadvantages, such as the possibility of compound degradation, undesirable reactions (caramelization and Maillard reaction) and the loss of selectivity [18]. Therefore, the main goal of this study was to evaluate the antioxidant compounds extracted from Salicornia through subcritical water extraction using different temperatures (110, 120, 140, 160 and 180 °C) and validate the most promising extract by in vitro and ex vivo assays as a potential cosmetic ingredient.

## 2. Materials and Methods

### 2.1. Chemicals

All chemicals were from Sigma-Aldrich (Buchs, Switzerland), Sigma Chemical Co. (St. Louis, MI, USA) and Merck KGaA (Darmstadt, Germany). Sodium phosphate monobasic was from Fluka Analytical (Darmstadt, Germany). The cell reagents were provided by Life Technologies, S.A. (Madrid, Spain) and Biowest (Nuaillé, France). Human foreskin fibroblasts (HFF-1) were obtained from ATCC (Manassas, VI, USA). Human immortalized nontumorigenic keratinocytes cell line (HaCaT) was acquired from CLS Cell Lines Service (Eppelheim, Germany). Elastatinal inhibitor and human neutrophil elastase were from Enzo Life Sciences (Farmingdale, NY, USA).

### 2.2. Sample

*S. ramosissima* J. Woods was kindly supplied in March 2021 by RiaFresh, located in Faro, Portugal. The sample was identified according to Flora Ibérica [19] by a botanist from the research team. Samples were dehydrated (Excalibur Food Dehydrator, Sacramento, CA, USA) at 41 °C for 24 h, milled in a grinder (Moulinex A320, Paris, France) to a particle size of 1 mm and, subsequently, kept at room temperature (20 °C) in the dark until further extraction.

### 2.3. Subcritical Water Extraction (SWE) of S. ramosissima

The extraction of *S. ramosissima* was conducted in a home-made subcritical batch-type extractor of 1.7 L, according to Švarc-Gajić et al. [20]. In order to determine the most efficient temperature to extract antioxidants from *S. ramosissima*, different temperatures (ranging from 110 °C to 180 °C) were tested, during a 30 min period, applying a pressure of 20 bars and maintaining a sample-to-solvent ratio of 1:20. The movements of the vibrational platform (3 Hz) were supported by housing the extraction vessel during the extraction process. Afterwards, a flow-through water bath (20 ± 2 °C) was used for cooling the extraction vessel, followed by depressurization through the valve opening. Afterwards, the extracts were filtered through Whatman n° 1 paper and frozen at −80 °C. Subsequently, the extracts were subjected to lyophilization (Telstar, model Cryodos −80, Barcelona, Spain) and stored at 4 °C. For the further experiments, the final residue was dissolved in distilled water. The extraction yield corresponding to the recovery of antioxidants was determined for each extract, resulting as the ratio between the total weight of the lyophilized extract and the total weight of the extract obtained after the SWE.

### 2.4. Quantification of the Bioactive Compounds

#### Determination of Total Polyphenols Content

The Total Phenolic Content (TPC) was determined according to the methodology described by Singleton et al. [21], with minor modifications. Briefly, the sample was dissolved in distilled water and an aliquot (30 µL), at concentrations ranging from 500 to 1000 μg/mL, was added to 150 µL of Folin–Ciocalteu’s reagent (1:10, *v*/*v*) and 120 µL of 7.5% (*w*/*v*) Na_2_CO_3_ solution in a 96-well microplate. Thereafter, the microplate was incubated in a microplate reader (Sinergy HT, BioTek Instruments, Inc., Winooski, VT, USA) for 15 min at 45 °C, followed by the measure of the absorbance at 765 nm. The calibration curve was prepared with gallic acid (linearity range: 5–100 mg/L, *R*^2^ = 0.999), working as standard. The results were expressed as milligrams of gallic acid equivalents (GAE) per gram of extract on dry weight (dw) (mg GAE/g dw).

### 2.5. In Vitro Antioxidant/Antiradical Activities

#### 2.5.1. Ferric Reducing Antioxidant Power Assay

The Ferric Reducing Antioxidant Power (FRAP) was evaluated according to Benzie and Strain [22], with minor modifications. The results were expressed as the concentration of antioxidants having a ferric-reducing ability equivalent to that of 1 mmol/L FeSO_4_, expressly in µM ferrous sulphate equivalents (FSE) per gram of extract on dw (µM FSE/g dw).

#### 2.5.2. ABTS^•+^ Radical Scavenging Activity Assay

The ABTS^•+^ radical-scavenging activity of samples was determined using the method of Re et al. [23]. The results were expressed in mg of ascorbic acid equivalents (AAE) per gram of extract on dw (mg AAE/g dw).

#### 2.5.3. DPPH^•^ Radical Scavenging Activity Assay

The ability to reduce the radical DPPH^•^ was assessed according to the procedure described by Barros et al. [24], with minor alterations. The results were expressed as mg of Trolox equivalents (TE) per gram of plant material on dw (mg TE/g dw).

### 2.6. Reactive Oxygen Species Scavenging Capacity Assays

#### 2.6.1. Superoxide Radical Scavenging Assay

The scavenging activity of *S. ramosissima* extracts was evaluated based on the reduction of NBT into diformazan influenced by O_2_^•−^, according to Gomes et al. [25]. The results were expressed as the inhibition, in IC_50_, of the NBT reduction to diformazan.

#### 2.6.2. Hypochlorous Acid scavenging Assay

The uptake ability of hypochlorous acid (HOCl) was determined based on the HOCl-induced oxidation of dihydrorhodamine 123 (DHR), according to the procedure described by Gomes et al. [25]. The results were expressed as the inhibition, in IC_50_, of HOCl-induced oxidation of DHR.

### 2.7. Phytochemical Profile of S. ramosissima Extracts

#### 2.7.1. Preparation of Polyphenols Standards

All 40 phenolic compounds were from Sigma-Aldrich and Extrasynthese, with a purity of at least ≥90%. Methanol and formic acid were gradient grade and obtained from Merck (Darmstadt, Germany). Individual standard solutions were prepared in HPLC grade methanol at concentration levels ranging from 1 to 5 g/L, being then used to prepare the mixtures of solutions for the calibration curves. 

#### 2.7.2. High Performance Liquid Chromatography (HPLC) System

HPLC analyses were carried out in a Shimadzu HPLC system (Shimadzu Corporation, Kyoto, Japan) equipped with an LC-20AD prominence pump, a DGU-20AS prominence degasser, a CTO-10AS VP column oven, an SIL-20A HT prominence autosampler, and an SPD-M20A photodiode array detector. A Gemini C18 column (250 mm × 4.6 mm, 5 μm) from Phenomenex (Torrance, CA, USA) was used and operated at 25 °C.

#### 2.7.3. HPLC Chromatographic Conditions

The phenolic composition of Salicornia subcritical water extracts was determined by HPLC, as previously described by Moreira et al., with some modifications [26]. Table S1 (Supplementary Material) reports the calibration data used for the quantification of the individual phenolic compounds. The results are reported as means ± standard deviations of triplicate independent analyses.

### 2.8. Cell Viability Assays

Two skin cell lines, fibroblasts (HFF-1) and keratinocytes (HaCaT), were employed to assess the cell viability effects of the Salicornia extracts. The cells were cultivated as previously reported by Pinto et al. [16]. Passages 9–10 and 82–83 were, respectively, used for HFF-1 and HaCaT cells. DMEM and Triton X-100 were used as positive and negative controls, respectively. The obtained results were expressed as percentages of cell viability.

### 2.9. Ex Vivo Permeation Assay in Franz Diffusion Cell

The ex vivo permeation assay was performed in Franz diffusion cells (PermeGear, Inc., Hellertown, PA, USA) with a diffusion area of 0.785 cm^2^ and receptor volume of 5 mL, and by using human skin as described by Rodrigues et al. [27]. The skin, acquired from an abdominal surgery from a healthy female volunteer (Department of Plastic Surgery, São João Hospital, Porto, Portugal), had a thickness of 0.8 mm. The protocol employed (protocol code: 90_17) was approved by the Bioethics Committee of the São João Hospital and the volunteer signed a written consent form. Prior to its usage in the permeation experiment, the skin was washed with ultra-pure water, and stripped of the fat layer, then the cleanest parts were selected, and stored at −20 °C. The prepared skin, previously hydrated with PBS for 10 min, was mounted between the two chambers (the donor and receptor chamber) of the Franz cell. The receptor chamber of the Franz cell was filled with 5 mL of PBS (pH 7.4). The solution present in the lower chamber was continuously stirred by a magnetic bar and maintained at 37 °C throughout the experiment. Afterwards, 500 µL of the diluted sample was added to the donor compartment. Samples of 200 µL were withdrawn from the receptor chamber at time intervals of 0 min, 30 min, 45 min, 1 h, 1 h 30, 2 h, 2 h 30, 3 h, 3 h 30, 4 h, 5 h, 6 h, 7 h, 8 h and 24 h. The same volume of PBS was immediately added to the receptor chamber. After 24 h, a sample from the donor chamber was collected and the portion of the skin used in the assay was transferred to eppendorfs (dermis and epidermis were separated). The assessment was executed in triplicate for each sample. The permeation was quantified by LC/DAD-ESI-MS, according to the methodology described by Silva et al. [28].

### 2.10. Enzymatic Assays

#### 2.10.1. Human Neutrophil Elastase Inhibition Assay

The extracts were evaluated regarding the elastase inhibition activity via human neutrophil elastase inhibition assay, based on the Enzo Life Science’s protocol for the neutrophil elastase colorimetric drug discovery kit. The results were expressed as the inhibition, in IC_5_ and IC_70_, of the human neutrophil elastase.

#### 2.10.2. Hyaluronidase Inhibition Assay

The hyaluronidase inhibitory activity of the extracts was investigated using the hyaluronidase inhibition assay, based on the Sigma-Aldrich’s protocol for the enzymatic assay of hyaluronidase (3.2.1.35) and Nema et al. [29]. The results were expressed as the inhibition, in IC_10_, of hyaluronidase.

### 2.11. Statistical Analysis

The results were reported as mean ± standard deviation of at least triplicate experiments or mean ± standard error of the mean of at least duplicate experiments. IBM SPSS Statistics 28.0 software (SPSS Inc., Chicago, IL, USA) was employed to perform the statistical analysis of the data. One-way ANOVA was applied to investigate the differences between the samples for all assays and post hoc comparisons of the means were performed with Tukey’s HSD test. A denoting significance was accepted for *p* < 0.05 in all cases. A correlation study was also performed, with the Pearson’s regression coefficient ‘*R*’ with *p*-value being selected.

## 3. Results and Discussion

### 3.1. Extraction Yield, TPC and Antioxidant/Antiradical Activities

The extraction yield, TPC and antioxidant/antiradical activities of the SWE extracts are summarized in Table 1.

The extraction yield ranged between 21.27% and 21.65%, for the 110 °C and 140 °C extracts, respectively. Silva et al. evaluated the *S. ramosissima* extracts prepared by MAE and conventional extraction (CE), namely maceration, and reported extraction yields between 21.14% and 26.10%, respectively [30]. The CE results were similar to the ones achieved in the present study; however, the MAE extracts demonstrated higher yields. In another study, Pinto et al. optimized the extraction of bioactive compounds from *S. ramosissima* biowaste by maceration through a mathematical model (Response Surface Methodology (RSM)) [31]. According to the authors, the extraction yields were similar to the ones obtained in the current study, achieving values around 30% [31].

Regarding the TPC, the lowest value obtained was 14.55 mg GAE/g dw and the highest 25.66 mg GAE/g dw, respectively, for the extracts prepared at 160 °C and 140 °C. Significant differences (*p* < 0.05) were observed between the extract obtained at 160 °C and the other extracts. Silva et al. reported values ranging between 8.34 and 15.02 GAE/g dw, respectively, for the *S. ramosissima* MAE and CE extracts, which are lower than the ones achieved in the present work [30]. The results presented in Table 1 were also higher than the ones reported by Lima et al. and ranged between 6.18 and 12.9 mg GAE/g dw [5]. In that experiment, the *S. ramosissima* extracts were prepared with 80% of acetone (1:40, *w/v*) aiming to screen the antioxidant activity of the plant at different levels of salinity [5]. The highest value obtained corresponded to the plant cultivated in 200 mM of salinity medium [5]. In another study, Barreira et al. prepared ethanolic extracts (1:40, *w*/*v*) of *S. ramosissima* by maceration and achieved a TPC of 33.00 mg GAE/g dw, a value superior to the one described in the present work [32]. Pinto et al., whose study was previously mentioned, also executed this assay, obtaining TPC values between 480.35 and 905.16 µg GAE/100 mg dw, which are inferior to the results reported in the current study [31].

The FRAP assay is based on the ability of antioxidants to reduce the ions present in a chromogenic ligand. Tripyridyltriazine (TPTZ), being a 2,4,6-tripyridyl-*s*-triazine complex (Fe(III)-TPTZ), reduced to a colored ferrous tripyridyltriazine (Fe(II)-TPTZ). As Table 1 shows, the extract prepared at 140 °C exhibited the highest value (418.56 µmol FeS/g), while the extract obtained at 120 °C achieved the lowest (137.26 µmol FeS/g), with significant differences among them (*p* < 0.05). These results were substantially higher than the ones reported by Silva et al. that varied between 60.61 and 65.56 µmol FeS/g, probably due to the different methodologies employed, as previously reported [30]. The results reported by Pinto et al. were also lower, ranging from 6.98 to 13.91 µmol FSE/100 mg [31]. In fact, different authors reported higher values of antioxidant activity when SWE was employed, comparated to conventional solvent extractions [33,34,35]. These differences can be explained not only by the different solvents used, but, most importantly, by the technique employed. The dissimilarity may also be due to the extraction time and temperature used. To the best of our knowledge, there are no studies that employed SWE to recover antioxidant compounds from this halophyte.

Concerning the ABTS^•+^ assay, the values ranged from 28.23 to 39.50 mg AAE/g dw, respectively, for the temperatures of 120 °C and 160 °C (*p* < 0.05). Once again, the results were significantly higher than the ones reported by Silva et al. that varied between 15.55 and 17.74 µg AAE/g dw [30]. Lima et al. also assessed the ABTS^•+^ scavenging capacity of this halophyte, presenting IC_50_ values between 2.12 and 4.44 mg/mL [5], while Pinto et al. obtained lower values that ranged from 773.24 to 1308.25 µg AAE/100 mg dw [31]. However, in this study, the extracts were not efficient enough in the sequestration of the ABTS^•+^ radical to assess the IC_50_. The percentage of inhibition (at the highest concentration tested) ranged from 29.58 to 38.99 %.

As shown in Table 1, the lowest DPPH^•^ scavenging value obtained was 10.17 mg TE/g dw (for the temperature of 110 °C), while the highest was 34.41 mg TE/g dw (for the temperature of 160 °C), presenting significant difference (*p* < 0.05). Most of the studies express the results in different units from the present work, except the one of Pinto et al. [31]. According to the authors, the DPPH^•^ results ranged from 145.85 to 1404.12 µg TE/100 mg dw, which are lower than the ones achieved in the current study [31]. Some studies assessed the scavenging capacity of this halophyte against DPPH^•^; however, the results were expressed as IC_50_. The percentage of inhibition (at the highest concentration tested) varied from 15.72 to 56.21%. The IC_50_ value from the sample extracted at 180 °C (824.57 µg/mL) was significantly higher than the positive control (89.80 µg/mL). 

Higher extraction temperatures seemed to extract more polyphenols, meaning that the antioxidants extracted from the halophyte must be moderately polar or weakly polar compounds. Therefore, the identification and quantification of phenolic compounds by HPLC was imperative for reliable conclusions.

### 3.2. Phytochemical Profile of S. ramosissima Extracts

**Figure 1:**
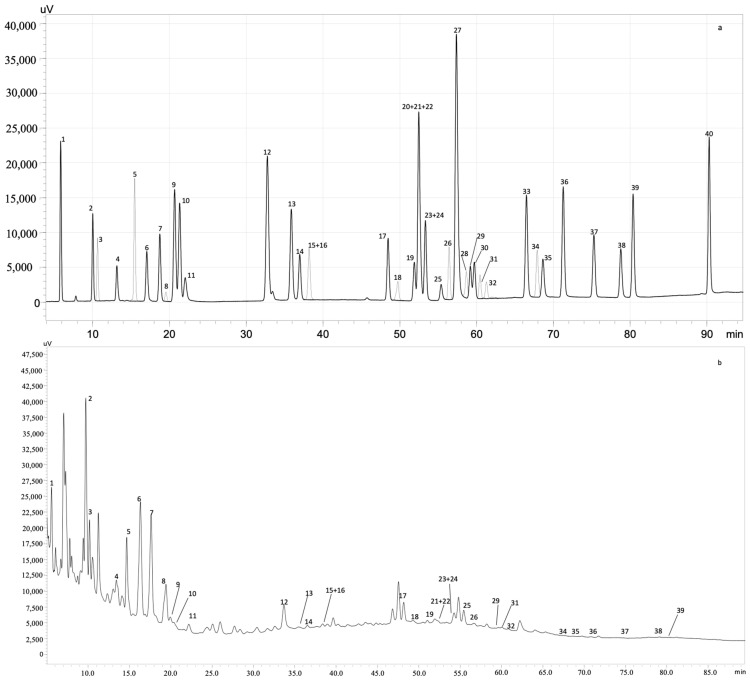
HPLC-PDA chromatogram monitored at 280 nm for (**a**) polyphenol standard mixture of 5 mg/L and (**b**) subcritical water extract obtained at 140 °C; peak identification: (1) gallic acid, (2) protocatechuic acid, (3) neochlorogenic acid, (4) (+)-catechin, (5) caftaric acid, (6) caffeine, (7) chlorogenic acid, (8) 4-*O*-caffeyolquinic acid, (9) vanillic acid, (10) caffeic acid, (11) syringic acid, (12) (−)-epicatechin, (13) *p*-coumaric acid, (14) *trans*-ferulic acid, (15) sinapic acid, (16) *trans*-polydatin, (17) naringin, (18) 3,5-di-caffeoylquinic acid, (19) quercetin-3-*O*-galactoside, (20) resveratrol, (21) quercetin-3-*O*-glucopyranoside, (22) rutin, (23) phloridzin, (24) ellagic acid, (25) 3,4-di-*O*-caffeoylquinic acid; (26) myricetin, (27) cinnamic acid, (28) quercitrin, (29) kaempferol-3-*O*-glucoside, (30) isorhamnetin-3-*O*-glucoside, (31) kaempferol-3-*O*-rutinoside, (32) isorhamnetin-3-*O*-rutinoside, (33) naringenin, (34) *trans*-epsilon viniferin, (35) quercetin, (36) phloretin, (37) tiliroside, (38) kaempferol, (39) apigenin and (40) chrysin.

The bioactive compounds of *S. ramosissima* extracts were identified and quantified by HPLC. The results are summarized in Table 2 and an example of an obtained chromatogram (from the halophyte’s extracted at 140 °C) is shown in Figure 1. 

The total amount of phenolic compounds quantified varied between the different extracts. The halophyte sample extracted at 180 °C presented the highest phenolic content (1739.28 mg/100 g dw), followed by the extracts prepared at 120 °C (1589.32 mg/100 g dw), 140 °C (1511.31 mg/100 g dw), 160 °C (1260.68 mg/100 g dw) and 110 °C (750.13 mg/100 g dw). These results are not in line with the TPC reported in the previous section, where the lowest and highest TPC obtained were, respectively, for the extracts prepared at 160 °C and 140 °C. These differences could be explained by reaction kinetics that occur in the spectrophotometric assays as well as the presence of interferents (such as sugars) or limitations of the spectrophotometric assays, resulting in inaccurate results.

According to Table 2, the main components extracted at all temperatures were phenolic acids (non-flavonoid) and flavanols (flavonoid). Regarding the extract obtained at 110 °C, these classes represented 42% and 37% of the total phenolic composition, respectively. However, for the extract obtained at 120 °C, these values were 51% and 30%, respectively. At 140 °C, 160 °C and 180 °C, the percentage of phenolic acids and flavanols were 53% and 27%, 62% and 14%, and 61% and 30%, respectively. Furthermore, as shown in Table 2, the phenolic acids were always present in higher quantity than flavanols, despite the temperatures. Theoretically, the increase in phenolic acid extraction with the temperature rise was expected (although not linear), since phenolic acids are habitually extracted with non-polar organic solvents, such as ethanol, methanol, and acetone [36]. Therefore, as the temperature of the subcritical water rises, the dielectric constant decreases to values close to those dielectric constants of common organic solvents, mimicking their extraction properties. Regarding flavonoids, since they are generally medium polar, better extraction was expected at higher temperatures. Cheng et al. proposed that the range between 150 and 200 °C (with times from 10 to 50 min) is optimal for the flavonoid SWE extraction [37], which seems to be in line with the general results of the present study (even though, at the temperature of 120 °C, the results were not far behind the ones achieved at 160 °C and 180 °C, and in some cases even surpassed those).

Concerning the phenolic acids, chlorogenic and protocatechuic acids were the main compounds identified in all extracts. Chlorogenic acid, which is the ester of the caffeic acid and quinic acid, is known by its carcinogenic and antimutagenic properties as well as its antioxidant activity and scavenging capacity of ROS [38]. Protocatechuic acid, a human metabolite of cyanidin-glucosides, is a natural phenolic compound also known to act as an antioxidant, among other roles, such as anti-inflammatory, antitumor, neuroprotective, antibacterial, anti-apoptotic and antidiabetic [39]. 

Regarding flavanols, catechin was the principal compound present in all extracts. Epicatechin was also detected, but only in the extracts prepared at 140 °C (49.61 mg/100 g dw) and 160 °C (12.29 mg/100 g dw). In both cases, the quantity of epicatechin did not exceed catechin. Catechin has well demonstrated antioxidant, antibacterial, anti-inflammatory, antitumor, antiviral, antidiabetic, and immunomodulatory properties [40].

Nevertheless, flavanones and flavonols were also identified and quantified, although in substantially smaller amounts, for all tested extraction temperatures. Regarding flavanones, only naringin was identified. Quercetin-3-*O*-galactoside was the major flavonol quantified. This flavonol acts as a ROS scavenger [41].

At all extraction temperatures, flavones were either not detected or found in quantities below the limit of quantification. Moreover, other phenolic compounds were quantified, i.e., phloridzin, phloretin and caffeine, with the last one being the most present.

Silva et al. also identified and quantified phenolic compounds in *S. ramosissima* extracts, although the extraction methods were different from the present study (namely, CE and MAE) [30]. In general, significantly fewer quantities of all classes were obtained when compared to the results of the current study, despite the phenolic compounds identified being similar [30]. However, phenolic acids and flavonols were the principal constituents of both extraction methods, in contrast with phenolic acids and flavanols that were predominant in the present study [30]. Furthermore, unlike the results of most of the extracts of the present study, flavones (0.0087 mg/g dw and 0.0069 mg/g dw for CE and MAE, respectively) were detected by Silva et al. [30]. Both studies identified and quantified phloridzin and phloretin [30]. Nonetheless, Silva et al. reported significantly lower values (0.0205 mg/g dw and 0.0178 mg/g dw for CE and MAE extracts, respectively) when compared to the results obtained in this experiment (ranging from 6.88 to 26.89 mg/100 g dw) [30]. Unlike the current study, trans-epsilon viniferin, caffeine and trans-polydatin were not detected in both extracts by Silva et al. [30].

Finally, since the sample extracted at 180 °C presented a higher phenolic content, along with a major phenolic acid predominance (previously described as good cosmetic ingredients [42]), it was selected for the next assays.

### 3.3. Reactive Oxygen Species Scavenging Capacity Assays

As previously stated, oxidative stress contributes to skin aging due to the higher levels of ROS, such as oxygen peroxide (H_2_O_2_) and superoxide anion radical (O_2_^•−^), that can generate oxidative damage in DNA, protein, and lipids. The results of the ROS scavenging capacity of the best extract are presented in Table 3.

Superoxide anion radical is the proximal mitochondrial ROS [43]. When it is produced by mitochondria, it leads to the formation of H_2_O_2_, resulting from the dismutation catalyzed by superoxide dismutase (SOD) [43]. Thus, the production of O_2_^•−^ seems to be the beginning of several reaction chains that may result in the assembly of more ROS species.

As shown in Table 3, the lowest O_2_^•−^ scavenging value was obtained by gallic acid, presenting an IC_50_ of 23.82 µg/mL, while the highest, with an IC_50_ of 158.87 µg/mL, is from the *S. ramosissima* extract. For both positive controls, lower IC_50_ values were obtained when compared to the halophyte extract. Significant differences (*p* < 0.05) were observed between the extract and the positive controls as well as between catechin and gallic acid. Silva et al. also assessed the O_2_^•−^ scavenging capacity of this halophyte, achieving a IC_50_ value of 979.36 µg/mL, which represents worse scavenging activity against O_2_^•−^ when compared to the value obtained in the current study [30]. The authors also reported the higher O_2_^•−^ scavenging values of gallic acid (101.37 µg/mL) and catechin (123.78 µg/mL) [30]. Furthermore, Pinto et al. reported an IC_50_ of 324.82 µg/mL, which is also a significantly lower capacity than the results from the present experiments. According to the authors, gallic acid and catechin IC_50_ values were 24.08 and 41.81 µg/mL, respectively [31]. Considering that low IC_50_ values correlate with high antioxidant activity, our results pointed to a better antioxidant performance. Gallic acid showed the highest capacity to quench O_2_^•−^, followed by catechin and the Salicornia extract.

HOCl is a powerful oxidizing agent, being one of the most reactive species generated from ROS [44]. Concerning the HOCl assay, catechin was the best scavenger (0.09 µg/mL), followed by gallic acid (0.61 µg/mL) and *S. ramosissima* extract (5.80 µg/mL). As Table 3 states, the extract IC_50_ value was higher than both positive controls. Significant differences (*p* < 0.05) were observed between the extract and the positive controls, but not between catechin and gallic acid. Silva et al. reported IC_50_ values for this matrix ranging from 90.28 to 104.64 µg/mL, which are significantly higher when compared to the ones achieved in this study [30]. Moreover, the authors additionally stated higher IC_50_ values for gallic acid (11.76 µg/mL) and catechin (0.96 µg/mL) [30]. When compared to the present study, Pinto et al. also obtained a higher IC_50_ for the optimal extract, corresponding to 27.61 µg/mL, followed by gallic acid (1.77 µg/mL) and catechin (0.38 µg/mL) [31]. As previously referenced, since a high antioxidant activity is described by a low IC_50_ value, our results remain, pointing out a superior antioxidant performance.

### 3.4. Cellular Viability Assays

To study the extract’s safety on skin cell lines, the effect of the extract was assessed on keratinocytes (HaCaT) and fibroblasts (HFF-1). The results are summarized in Table 4 and Table 5.

As shown in Table 4, after the exposure of the fibroblasts to 10, 100, 1000 μg/mL of extract, the cellular viability decreased to 86.15, 89.19, 86.11%, respectively. On the other hand, at 0.1 and 1 μg/mL, the viability was 97.39 and 100.81%, respectively. No significant differences (*p* > 0.067) were observed between the different concentrations tested.

Regarding HaCaT, the *S. ramosissima* extract did not have a negative effect on the cell viability (Table 5). Similarly, no significant differences (*p* > 0.059) between the different concentrations tested were observed.

Pinto et al. assessed the cellular viability of skin cells (keratinocytes and fibroblasts) after exposure to the optimal *S. ramosissima* by-product extract [31], reporting that the extract was safe in all tested concentrations (0.1–1000 μg/mL). According to the authors, the keratinocytes’ viability ranged between 93.98% and 108.63%, respectively, for the concentrations of 1000 μg/mL and 0.1 μg/mL, while the fibroblasts’ viability was between 93.51% and 98.92%, respectively, after exposure to 0.1 μg/mL and 1000 μg/mL [31]. In another study, Silva et al. evaluated the cellular viability of intestinal cell lines (Caco-2 and HT29-MTX) [30]. According to the authors, the HT29-MTX cell line viability did not decrease after exposure to all tested concentrations (0.1–1000 μg/mL). For the concentration of 1000 µg/mL, the viability was 97.04% and 94.32%, for the CE for MAE extracts, respectively [30]. Furthermore, the Caco-2 viability did not decrease after exposure to any of the CE extracts concentrations; however, at the highest concentration of the MAE extract, the viability decreased to 86.55% [30]. 

Shin et al. studied the protective effects of halophytes (*Limonium tetragonum*, *Triglochin maritimum*, *Artemisia scoparia*) and red ginseng complex against UV-induced skin damage. The complex was proportioned as the following: *L. tetragonum* 2: *A. scoparia* 1: *T. maritimum* 1: *Red ginseng* 2 and ethanol 50%, with a 20:1 ratio of extraction [45]. The authors evaluated the viability of HaCaT cells after exposure to the complex and observed that the keratinocytes’ viability was high (approximately 85%) [45]. As can be observed, the viability achieved in the present study was higher than the values reported by Shin et al.

### 3.5. Ex Vivo Permeation Assays in Franz Diffusion Cells

Assessing the permeation of polyphenols is a key step when studying the potential of new cosmetic ingredients, since their function can be compromised if permeation through the skin layers is not efficient. Biological (e.g., age, metabolism) and physiochemical (e.g., size, molecular weight, polarity) properties of the compounds influence their transfer to desirable skin layers. On that note, a Franz permeation assay was performed. Table 6 summarized the permeation of the principal phenolic compounds. After deep analysis, three major phenolic compounds were identified in the receptor chamber: rutin (*m/z* = 609), quercetin (*m/z* = 301) and syringic acid (*m/z* = 197).

Rutin is usually found in plant extracts and displays antioxidant [46], anti-inflammatory [47], antidiabetic, anti-cancer, anti-aging properties [46] and antibacterial properties [48], along with providing cytoprotective effects against radiation [47]. It can interact intracellularly with molecules from the antioxidant system, restoring the low-molecular weight antioxidant pool and, furthermore, inducing the rise of antioxidant enzyme activity, while upregulating cytoprotective genes [48]. Despite all the aforementioned characteristics, rutin has some drawbacks, such as low stability and bioavailability [49]. Nonetheless, this molecule is proven to engage well in protecting the skin against UVB irradiation-induced skin damage when dissolved in a formula [46].

Quercetin (3,3′,4′,5,7-pentahydroxyflavone or (2-(3,4-dihydroxyphenyl)-3,5,7-trihydroxy-4Hchromen-4-one) is a polyphenol often identified in green and black tea, apples, onions, and red grapes [50]. This molecule presents antioxidant, anti-inflammatory [50], anticancer [50,51], anti-aging [51], antibacterial and antiviral effects [52]. However, quercetin is also poorly soluble in water (which hinders its skin absorption and overall efficacy) and external factors such as light, pH and temperature can compromise its stability, resulting in low bioavailability [50].

To the best of our knowledge, there are not many studies regarding the effects of syringic acid in the skin. However, according to few of them, this acid (*O*-methylatedtrihydroxybenzoic acid or 4-hydroxy-3,5-dimethoxybenzoic acid) can be part of oils, wine, edible fruits, and plants’ composition [53], displaying antioxidant, anti-inflammatory [54], anti-endotoxic, hepatoprotective [53] and anticancer properties [55].

Results showed that quercetin is the principal phenolic compound that permeated the skin after 0.5 h (0.029 μg/mg dw), followed by rutin (0.017 μg/mg dw) and syringic acid (0.015 μg/mg dw). That outcome was expected, since quercetin is the least water-soluble polyphenol (0.01 mg/mL), while syringic acid has the highest hydrophilic power (5.78 mg/mL). More hydrophobic molecules should permeate better due to the lipophilic nature of the outer *Stratum corneum*.

After 8 h, the rutin (0.047 μg/mg) and syringic acid (0.052 μg/mg dw) permeation rate began to decrease, whereas quercetin (0.091 μg/mg dw) increased exponentially, followed by a decrease at the time point 24 h (0.087 μg/mg dw). At the end of the assay, the three phenolic compounds permeated to a lesser extent. Regarding the epidermis, the skin’s first layer, quercetin was the principal compound identified (0.022 μg/mg dw), closely followed by syringic acid (0.020 μg/mg dw) and rutin (007 μg/mg dw), significantly far behind. However, rutin was not detected in the dermis. Quercetin and syringic acid were found in the aforesaid region, accounting for 0.012 μg/mg dw and 0.016 μg/mg dw of permeation, respectively. The overall scenario dictates that 1% of rutin, 5% of quercetin and 3% of syringic acid permeated through the epidermis, although only 3% of quercetin and syringic acid were able to reach dermis. 

To the best of our knowledge, this is the first study that performed ex vivo permeation analysis on *S. ramosissima* extracts.

### 3.6. Hyaluronidase and Elastase Inhibition Assays

The skin exposure to photoaging factors that lead to ROS accumulation can activate the dermal enzymes, including collagenase, elastase, and hyaluronidase, which are enrolled in the degradation of collagen, elastin, and hyaluronic acid, respectively [56]. These enzymes play a fundamental role in skin flexibility, integrity, and elasticity, resulting in a healthier and younger aspect [56]. Consequently, their inhibition may maintain skin youth and elasticity, retarding aging, being one of the most successful strategies. Elastin, an elastic protein of the connective tissue [57], plays an important role in the prevention of wrinkles, skin firmness and elasticity [57]. The levels of elastin naturally decline with age, inducing the development of wrinkles and skin sagging [57]. Alternatively, hyaluronic acid, also known as hyaluronan, promotes skin rejuvenation and resilience [56] by increasing viscosity and reducing the permeability of extracellular fluid [56]. This glucose-based polymer has great water-holding capability, preserving the skin moisture [56,58], inducing higher smoothness and decreasing the appearance of skin wrinkles [56]. During human aging, the hyaluronic acid content naturally decreases, leading to a decrease in skin strength, flexibility, and moisture [56]. The data in the literature states that plant derived polyphenols are remarkably strong elastase and hyaluronidase inhibitors [58]. 

Regarding the human neutrophil elastase (HNE) inhibition assay, an IC_5_ of 760 µg/mL was determined for the *S. ramosissima* sample. The IC_70_ calculated for elastatinal (positive control), which naturally inhibits the enzyme, was 51.26 µg/mL, which was significantly better. This is the first study that evaluated the capacity of *S. ramosissima* to inhibit elastase. However, there are studies concerning other halophytes, including *S. europae*, and their HNE-inhibiting behavior. For instance, Jiratchayamaethasakul et al. reported results regarding 70% ethanol extracts of 22 halophytes and their HNE inhibition [56]. The values (in percentage of elastase inhibition) ranged from 0.09% to 74.88% and the concentration of tested samples was 1 mg/mL [56]. *S. europaea* had the strongest anti-elastase activity among all tested halophytes [56]. Despite promising results, the authors selected a less eco-friendly conventional extraction procedure (with ethanol). In another study, Ahn et al. evaluated the antioxidant, antiwrinkle, and whitening activity of *S. bigelovii* [59]. The extraction procedure was performed with ethyl acetate [59]. At 20 and 100 ug/mL, the elastase inhibition activities were 2.9 and 32.5%, respectively [59]. Thus, *S. bigelovii* revealed a better inhibitory capacity of elastase; however, it is important to note that an environmentally unfriendly technique was employed. As previously stated, for cosmetic purposes, bioactive compounds should be extracted by eco-friendly approaches to meet the consumers’ expectations and compliance with regulations.

Concerning hyaluronidase inhibition, an IC_10_ of 64 µg/mL was determined for the *S. ramosissima* sample, while the positive control employed (epicatechin) showed a superior inhibition capacity (IC_10_ = 39.4 µg/mL). As far as we know, this is the first study that evaluated the hyaluronidase inhibition of *S. ramosissima*. In the study of Jiratchayamaethasakul et al. [56], among all tested halophytes, *S. europae* extract achieved the strongest anti-hyaluronidase activity [56].

## 4. Conclusions

The present study aimed to extract bioactive compounds from *S. ramosissima* through subcritical water extraction, in order to obtain a new cosmetic ingredient. The SWE extractions were performed at five temperatures (110, 120, 140, 160 and 180 °C) and the TPC and antioxidant/antiradical activities were evaluated to select the best extract. The extraction performed at 180 °C achieved the highest phenolic content, followed by the extracts prepared at 120 °C, 140 °C, 160 °C and 110 °C. The principal phenolic compounds extracted at all temperatures were phenolic acids and flavanols. Considering the phenolic content, the extract prepared at 180 °C was selected for further experiments. Regarding the ROS scavenging capacity, the *S. ramosissima* best extract achieved an IC_50_ = 5.80 µg/mL for the HOCl and an IC_50_ = 158.87 µg/mL for the O_2_^•−^ scavenging capacity. The cell viability assays showed that keratinocytes and fibroblasts were not affected after exposure to the extract (0.1–1000 µg/mL), proving its safety. The permeation of rutin, quercetin and syringic acid after 24 h was 1, 5 and 3%, respectively. Concerning the elastase inhibition assay, the IC_5_ determined was 760 µg/mL, while for the hyaluronidase inhibition assay, the IC_10_ achieved was 64 µg/mL. Epicatechin and gallic acid were used as positive controls for the hyaluronidase assay, obtaining an IC_10_ of, respectively, 34.9 and 24 µg/mL. The positive control used for the elastase assay was elastatinal, which attained an IC_70_ of 51.26 µg/mL. These results support the bioactivity of the *S. ramosissima* extract and its possible use as cosmetic ingredient with powerful skin antiaging properties. Regarding future perspectives, it is expected that the extract irritation potential be evaluated through a patch test and an extract-based cosmetic formulation be produced with the incorporated extract as an active ingredient.

## Figures and Tables

**Table 1:** Extraction yield, total phenolic content (TPC) and in vitro antioxidant/antiradical activities (evaluated by FRAP, ABTS^•+^ and DPPH assays) of *S. ramosissima* extracts obtained by SWE. Values are expressed as mean ± standard deviation (*n* = 3). Different letters in the same column indicate significant differences (*p* < 0.05).

Extraction Temperature (°C)	Extraction Yield (%)	TPC (mg GAE/g dw)	FRAP (µmol FSE/g dw)	ABTS^•+^ (mg AAE/g dw)	DPPH (mg TE/g dw)
110	21.27 ± 0.14	22.41 ± 0.04 ^a^	193.32 ± 7.72 ^c^	31.86 ± 3.28 ^b^	10.17 ± 1.67 ^c^
120	21.34 ± 0.07	23.86 ± 2.05 ^a^	144.37 ± 10.05 ^d^	28,23 ± 0.92 ^b^	19.93 ± 2.19 ^b,c^
140	21.65 ± 0.24	25.66 ± 2.22 ^a^	422.57 ± 5.67 ^a^	32.72 ± 3.27 ^a,b^	23.12 ± 1.77 ^a,b,c^
160	21.42 ± 0.01	14.55 ± 2.04 ^b^	283.63 ± 1.17 ^b^	39.50 ± 3.95 ^a^	34.41 ± 3.63 ^a^
180	21.36 ± 0.05	21.53 ± 2.08 ^a^	264.95 ± 18.79 ^b^	34.97 ± 4.48 ^a,b^	28.14 ± 0.81 ^a,b^

**Table 2:** Identification and quantification of the bioactive compounds present in *S. ramosissima* extracted at 110 °C, 120 °C, 140 °C, 160 °C and 180 °C. Results were expressed as mean ± standard deviations (mg of compound/100 g dw).

Compounds	SWE 110 °C (mg/100 g dw)	SWE 120 °C (mg/100 g dw)	SWE 140 °C (mg/100 g dw)	SWE 160 °C (mg/100 g dw)	SWE 180 °C (mg/100 g dw)
**Phenolic acids**					
Gallic acid	37.88 ± 1.89	39.40 ± 1.97	42.90 ± 2.15	3.28 ± 0.16	94.01 ± 4.70
Protocatechuic acid	80.82 ± 4.04	197.64 ± 9.88	241.64 ± 12.08	139.16 ± 6.96	302.06 ± 15.10
Neochlorogenic acid	5.33 ± 0.27	40.72 ± 2.04	39.78 ± 1.99	39.10 ± 1.95	92.32 ± 4.62
Vanillic acid	12.24 ± 0.61	18.25 ± 0.91	36.95 ± 1.85	37.27 ± 1.86	36.47 ± 1.82
Caffeic acid	<LOQ	6.72 ± 0.34	9.69 ± 0.48	32.45 ± 1.62	51.83 ± 2.59
Syringic acid	4.11 ± 0.21	10.05 ± 0.50	10.56 ± 0.53	12.47 ± 0.62	12.00 ± 0.60
Caftaric acid	7.46 ± 0.37	131.11 ± 6.56	87.44 ± 4.37	78.08 ± 3.90	108.14 ± 5.41
Chlorogenic acid	135.35 ± 6.77	326.52 ± 16.33	270.77 ± 13.54	270.11 ± 13.51	238.37 ± 11.92
4-*O*-caffeyolquinic acid	11.55 ± 0.58	9.23 ± 0.46	11.91 ± 0.60	129.63 ± 6.48	105.47 ± 5.27
*p*-Coumaric acid	16.27 ± 0.81	31.16 ± 1.56	29.94 ± 1.50	23.70 ± 1.19	<LOD
Ferulic acid	1.30 ± 0.06	<LOD	<LOQ	<LOQ	<LOQ
Sinapic acid	<LOQ	ND	<LOQ	<LOQ	<LOQ
3.5-di-caffeoylquinic acid	<LOQ	<LOQ	8.84 ± 0.44	9.46 ± 0.47	<LOQ
Ellagic acid	<LOQ	<LOQ	<LOQ	<LOQ	<LOD
4.5-di-*O*-caffeoylquinic acid	4.42 ± 0.22	7.29 ± 0.36	7.26 ± 0.36	11.35 ± 0.57	14.09 ± 0.70
Cinnamic acid	ND	ND	ND	ND	ND
**∑Phenolic acids**	316.72	818.10	797.70	786.07	1054.77
**Flavanols**					
Catechin	277.13 ± 13.86	475.49 ± 23.77	359.43 ± 17.97	169.61 ± 8.48	519.93 ± 26.00
Epicatechin	ND	ND	49.61 ± 2.48	12.29 ± 0.61	ND
∑Flavanols	277.13	475.49	409.04	181.90	519.93
Flavanones					
Naringin	3.36 ± 0.17	9.80 ± 0.49	3.26 ± 0.16	7.65 ± 0.38	7.84 ± 0.39
Naringenin	ND	ND	ND	<LOD	ND
∑Flavanones	3.36	9.80	3.26	7.65	7.84
Flavonols					
Quercetin-3-*O*-galactoside	10.76 ± 0.54	23.51 ± 1.18	18.66 ± 0.93	51.64 ± 2.58	17.01 ± 0.85
Rutin	ND	ND	<LOQ	<LOQ	ND
Myricetin	14.20 ± 0.71	26.73 ± 1.34	<LOQ	<LOD	<LOQ
Kaempferol-3-*O*-glucoside	ND	<LOQ	<LOQ	<LOQ	<LOD
Kaempferol-3-*O*-rutinoside	3.17 ± 0.16	<LOQ	<LOQ	2.73 ± 0.14	ND
Quercetin	<LOD	<LOQ	<LOQ	<LOQ	ND
Tiliroside	ND	ND	<LOQ	<LOQ	ND
Kaempferol	<LOQ	<LOD	<LOD	<LOQ	<LOD
Quercetin-3-*O*-glucopyranoside	<LOQ	<LOD	<LOD	<LOD	<LOD
Isorhamnetin-3-*O*-glucoside	ND	ND	ND	ND	ND
Isorhamnetin-3-*O*-rutinoside	<LOD	<LOQ	<LOQ	4.97 ± 0.25	9.14 ± 0.46
**∑Flavonols**	28.13	50.24	18.66	59.34	26.15
Flavones					
Apigenin	ND	<LOD	<LOD	ND	<LOD
Chrysin	<LOD	<LOD	ND	ND	<LOD
Quercitrin	ND	ND	ND	ND	ND
**∑Flavones**	0.00	0.00	0.00	0.00	0.00
**Others**					
Phloridzin	6.88 ± 0.34	16.77 ± 0.84	19.47 ± 0.97	26.89 ± 1.34	21.80 ± 1.09
Phloretin	<LOQ	<LOQ	<LOQ	<LOQ	<LOD
Resveratrol	ND	ND	ND	ND	ND
trans-epsilon viniferin	<LOQ	<LOQ	<LOD	<LOQ	<LOQ
Caffeine	117.90 ± 5.89	218.91 ± 10.95	263.17 ± 13.16	198.83 ± 9.94	108.78 ± 5.44
trans-polydatin	<LOQ	<LOQ	<LOQ	<LOQ	<LOD
**∑Others**	124.77	235.69	282.64	225.72	130.58

ND: Not detected; LOQ: Limit of quantification; LOD: Limit of detection.

**Table 3:** Superoxide anion radical (O_2_^•−^) and hypochlorous acid (HOCl) scavenging capacities of the best *S. ramosissima* extract. Values are expressed as mean ± standard error of the mean (*n* = 3). Different letters in the same column indicate significant differences (*p* < 0.05).

Samples	O_2_^•−^	HOCl
	IC_50_ (µg/mL)	
*S. ramosissima* extract	158.87 ± 11.96 ^a^	5.80 ± 0.40 ^a^
Positive controls		
Gallic acid	23.82 ± 0.81 ^c^	0.61 ± 0.08 ^b^
Catechin	84.40 ± 4.58 ^b^	0.09 ± 0.01 ^b^

**Table 4:** Effects of the best *S. ramosissima* extract exposure on the viability (%) of HFF-1 cell line at different concentrations, as measured by the MTT assay. Values are expressed as mean ± standard deviation (*n* = 3).

Sample			Concentration (μg/mL)		
	0.1	1	10	100	1000
***S. ramosissima*** **extract**	97.39 ± 13.91	100.81 ± 14.86	86.15 ± 13.36	89.19 ± 18.59	86.11 ± 20.06
**Negative control**	0.00 ± 0.33				
**Positive** **control**	95.55 ± 9.09				

**Table 5:** Effects of *S. ramosissima* extract exposure on the viability (%) of HaCaT cell line at different concentrations, as measured by the MTT assay. Values are expressed as mean ± standard deviation (*n* = 3).

Sample		Concentration (μg/mL)			
	0.1	1	10	100	1000
***S. ramosissima*** **extract**	99.41 ± 8.88	100.02 ± 5.00	101.27 ± 5.09	101.25 ± 4.66	107.05 ± 7.34
**Negative control**	0.00 ± 0.18				
**Positive** **control**	102.79 ± 9.54				

**Table 6:** Permeation of *S. ramosissima* phenolic compounds, concretely identified as rutin, quercetin and syringic acid. Values are expressed as compound’s permeation ± standard deviation (*n* = 3).

	Compounds Permeation (%)			Compounds Permeation (μg/mg dw)		
Time (H)	Rutin	Quercetin	Syringic Acid	Rutin	Quercetin	Syringic Acid
**0.5**	3	7	2	0.017 ± 0.001	0.029 ± 0.001	0.015 ± 0.001
**1**	5	6	7	0.032 ± 0.002	0.026 ± 0.001	0.042 ± 0.02
**2**	6	10	9	0.041 ± 0.002	0.043 ± 0.002	0.056 ± 0.003
**4**	8	14	10	0.052 ± 0.003	0.058 ± 0.003	0.064 ± 0.003
**8**	7	21	8	0.047 ± 0.002	0.091 ± 0.005	0.052 ± 0.003
**24**	11	20	11	0.073 ± 0.004	0.087 ± 0.004	0.074 ± 0.004
**Epidermis**	1	5	3	0.007 ± 0.000	0.022 ± 0.001	0.020 ± 0.001
**Dermis**	-	3	3	-	0.012 ± 0.001	0.016 ± 0.001

## Data Availability

Data are contained within the article and supplementary materials.

## Supplementary Materials

The following supporting information can be downloaded at: https://www.mdpi.com/article/10.3390/antiox11122449/s1, Figure S1: HPLC-PDA chromatograms monitored at 280 nm for *S. ramosissima* extracts obtained at 110 °C (pink line), at 120 °C (blue line), at 140 °C (brown line), at 160 °C (green line) and at 180 °C (dark blue line); Table S1: Calibration data used for the quantification of individual phenolic compounds in salicornia subcritical water extracts.
