# Supplementary material for: Salicornia ramosissima: A New Green Cosmetic Ingredient with Promising Skin Effects
Source: Antioxidants (Basel). 2022 Dec 12;11(12):2449. doi: 10.3390/antiox11122449 (PMC9774727; doi:10.3390/antiox11122449)
Supplement: Supplementary file 1 [file antioxidants-11-02449-s001.zip › antioxidants-2049835-supplementary.pdf]

**Table S1.** Calibration data used for the quantification of individual phenolic compounds in salicornia subcritical water extracts

| Peak identification | Compounds | (m ± Δm)[a] | (b ± Δb)[b] | $r^2$ | LOD[c] (mg/L) | LOQ[d] (mg/L) |
|---|---|---|---|---|---|---|
| | **Phenolic acids** | | | | | |
| **1** | Gallic acid | 54220 ± 97 | 57.7 ± 6 | 0.9999 | 0.120 | 0.400 |
| **2** | Protocatechuic acid | 32064 ± 24 | 1024 ± 477 | 0.9999 | 0.104 | 0.346 |
| **3** | Neochlorogenic acid | 50502 ± 344 | 5751 ± 288 | 0.9998 | 0.449 | 1.495 |
| **5** | Caftaric acid | 18700 ± 50 | 966 ± 48.3 | 0.9999 | 0.177 | 0.591 |
| **7** | Chlorogenic acid | 27244 ± 57 | -1445 ± 144 | 0.9999 | 0.292 | 0.972 |
| **8** | 4-*O*-caffeyolquinic acid | 12334 ± 202 | -1032 ± 52 | 0.9995 | 0.19 | 0.632 |
| **9** | Vanillic acid | 36254 ± 55 | 1215 ± 121 | 0.9999 | 0.215 | 0.715 |
| **10** | Caffeic acid | 69190 ± 45 | 124 ± 12 | 0.9999 | 0.090 | 0.302 |
| **11** | Syringic acid | 61262 ± 72 | 2088 ± 209 | 0.9999 | 0.165 | 0.551 |
| **13** | *p*-coumaric acid | 146520 ± 1176 | -33763 ± 3376 | 0.9991 | 1.70 | 5.68 |
| **14** | *trans*-Ferulic acid | 61448 ± 39 | 1501.6 ± 789 | 0.9999 | 0.089 | 0.298 |
| **15** | Sinapic acid | 29742 ± 16 | 124 ± 12 | 0.9999 | 0.075 | 0.25 |
| **18** | 3,5-di-*O*-caffeoylquinic acid | 62289 ± 829 | -11003 ± 3153 | 0.9991 | 0.341 | 1.13 |
| **24** | Ellagic acid | 44947 ± 1040 | -30767 ± 20152 | 0.9973 | 3.09 | 10.3 |
| **25** | 3,4-di-*O*-caffeoylquinic acid | 28503 ± 410 | 32 ± 3 | 0.9991 | 0.383 | 1.27 |

| Peak identification | Compounds | (m ± Δm)[a] | (b ± Δb)[b] | $r^2$ | LOD[c] (mg/L) | LOQ[d] (mg/L) |
|---|---|---|---|---|---|---|
| **27** | Cinnamic acid | 183447 ± 103 | 2472 ± 247 | 0.9999 | 0.079 | 0.263 |
| | **Flavanols** | | | | | |
| **4** | Catechin | 13826 ± 56 | 3021 ± 1127 | 0.9999 | 0.568 | 1.89 |
| **12** | Epicatechin | 15117 ± 60 | 1178 ± 118 | 0.9999 | 0.558 | 1.86 |
| | **Flavanones** | | | | | |
| **17** | Naringin | 36536 ± 25 | 1384 ± 499 | 0.9999 | 0.095 | 0.317 |
| **33** | Naringenin | 68101 ± 90 | -2516 ± 1813 | 0.9999 | 0.186 | 0.619 |
| | **Flavonols** | | | | | |
| **19** | Quercetin-3-*O*-galactoside | 23597 ± 27 | 466 ± 47 | 0.9999 | 0.159 | 0.531 |
| **21** | Quercetin-3-O-glucopyranoside | 48964 ± 119 | 17.0 ± 0.9 | 0.9999 | 0.161 | 0.535 |
| **22** | Rutin hydrate | 83207 ± 51 | 1937 ± 1034 | 0.9999 | 0.087 | 0.289 |
| **26** | Myricetin | 27506 ± 260 | -12304 ± 5242 | 0.995 | 1.32 | 4.43 |
| **28** | Quercitrin | 32796 ± 199 | 2974 ± 149 | 0.9999 | 0.399 | 1.331 |
| **29** | Kaempferol-3-*O*-glucoside | 22171 ± 45 | 792 ± 79 | 0.9999 | 0.286 | 0.955 |
| **30** | Isorhamnetin-3-O-glucoside | 31445 ± 101 | 2000 ± 100 | 0.9999 | 0.211 | 0.702 |
| **31** | Kaempferol-3-*O*-rutinoside | 24228 ± 55 | -1094 ± 109 | 0.9999 | 0.319 | 1.06 |
| **32** | Isorhamnetin-3-O-rutinoside | 23574 ± 115 | 2374 ± 119 | 0.9999 | 0.323 | 1.075 |

| Peak identification | Compounds | (m ± Δm)[a] | (b ± Δb)[b] | $r^2$ | LOD[c] (mg/L) | LOQ[d] (mg/L) |
|---|---|---|---|---|---|---|
| **35** | Quercetin | 30017 ± 212 | -7386 ± 739 | 0.9997 | 0.992 | 3.30 |
| **37** | Tiliroside | 67386 ± 95 | -1771 ± 177 | 0.9999 | 0.198 | 0.658 |
| **38** | Kaempferol | 33473 ± 102 | -3301 ± 330 | 0.9999 | 0.430 | 1.43 |
| | **Flavones** | | | | | |
| **39** | Apigenin | 100189 ± 353 | -7277 ± 727 | 0.9999 | 0.495 | 1.65 |
| **40** | Chrysin | 88535 ± 143 | -3131 ± 313 | 0.9999 | 0.226 | 0.755 |
| | **Stilbenes** | | | | | |
| **16** | *trans*-Polydatin | 64539 ± 221 | 4280 ± 214 | 0.9999 | 0.226 | 0.753 |
| **20** | Resveratrol | 181186 ± 1303 | -16059 ± 803 | 0.9998 | 0.474 | 1.579 |
| **34** | *trans*-ε-viniferin | 61705 ± 529 | -1021 ± 51 | 0.9997 | 0.564 | 1.88 |
| | **Chalcones** | | | | | |
| **23** | Phloridzin dehydrate | 47726 ± 42 | 78 ± 8 | 0.9999 | 0.125 | 0.416 |
| **36** | Phloretin | 71734 ± 154 | 84 ± 8 | 0.9999 | 0.302 | 1.01 |
| | **Others** | | | | | |
| **6** | Caffeine | 54054 ± 257 | 6136 ± 307 | 0.9999 | 0.313 | 1.043 |

[a]m: slope ± standard deviation (n = 5) expressed in μV min/mg L; [b]b: intercept ± standard deviation (n = 5) expressed in μV min; [c]LOD: limit of detection; [d]LOQ: limit of quantification.

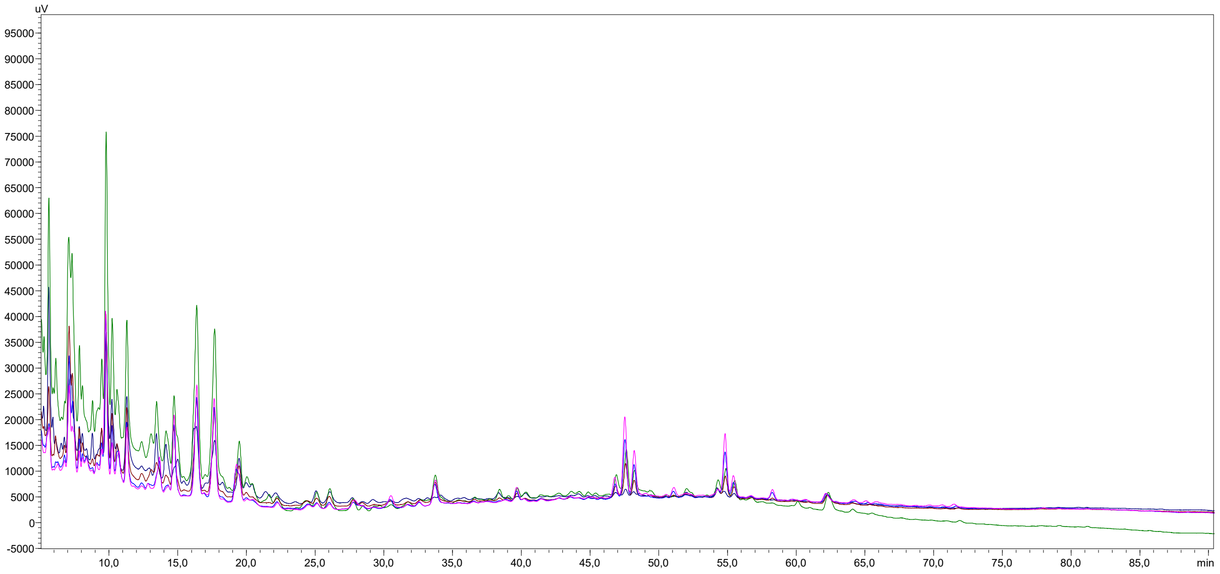


**Figure S1.** HPLC-PDA chromatograms monitored at 280 nm for *S. ramosissima* extracts obtained at 110 °C (pink line), at 120 °C (blue line), at 140 °C (brown line), at 160 °C (green line) and at 180 °C (dark blue line).
